# Hospital Expenditure.—The Commissariat

**Published:** 1896-07-18

**Authors:** 


### The Institutional Workshop.

### HOSPITAL EXPENDITURE.—THE COMMISSARIAT.

### XX.—THE DIGESTIBILITY OF FOREIGN MEAT.

The economical point of view is not the first for the hospital to take into consideration. The supply of meat for the patients must be regarded as a valuable part of their treatment, and it is of the highest importance that the four or six ounces which is prescribed should be easy of digestion and as rich as possible in food value. Moreover, it must be palatable, for the sick man not only will not, but cannot, force himself to eat what is distasteful.

It is not easy to gauge the actual food value of any kind of meat on account of the variations which soon become apparent between one animal and another, and between different parts of the same animal. The following table by König, showing the composition of mutton, reveals a very striking difference, for instance, between leg and shoulder:—

| | Water. | Albuminates. | Fat. |
|---|---|---|---|
| Hind-quarter of mutton ... | 41·97 ... | 14·39 ... | 43·47 |
| Shoulder ... ... ... | 60·38 ... | 14·57 ... | 23·62 |

Again, taking the same parts of different animals, König found that an even greater discrepancy arose from variations in feeding, and that according to the degree of fat present in the meat the amount of water increased and the amount of albuminates decreased.

| | Water. | Albuminates. | Fat. |
|---|---|---|---|
| Moderately fat... ... ... | 75·99 ... | 18·11 ... | 5·77 |
| Very fat... ... ... ... | 47·91 ... | 14·80 ... | 36·39 |

An effort was made in the following experiments to procure meat of the same description, but it was impossible to balance with any accuracy the exact condition of the animals. The three kinds of mutton selected for comparison were London-killed Scotch, at 10d. per lb.; frozen Canterbury, at 8d. per lb.; and frozen Australian, from the merino sheep, at 4d. per lb. Under the directions of Dr. Rideal, D.Sc.Lond., F.I.C., who conducted the experiments, all the lean meat, exclusive of skin, tendons, &c., was removed from a leg of each of the above kinds of mutton, and was then passed uncooked through a mincing machine. Samples of these meats were then submitted by him to artificial digestion experiments, with the view of determining whether the effect of freezing is to hinder or retard the digestibility of the meat. His report is as follows:—

"Twenty grams of the minced meat were digested for one hour at 38° C. with 20 c.c. of a 0·5 per cent. solution of pepsin (1 in 4,000), to which was added 1 c.c. decinormal hydrochloric acid and 50 c.c. distilled water. The experiments were carried out in duplicate, and the results obtained are summarised in the following table:—

| | Scotch. | Australian. | New Zealand. |
|---|---|---|---|
| Water per cent. ... ... | 74·25 ... | 72·45 ... | 71·32 |
| Nitrogen contents of dry meat | 12·38 ... | 11·55 ... | 11·39 |
| Percentage of dry meat digested in one hour— | | | |
| Exp. 1 ... ... | 41·2 ... | 37·4 ... | 37·2 |
| Exp. 2 ... ... | 43·3 ... | 38·6 ... | 37·3 |
| Mean ... ... ... | 42·25 ... | 38·0 ... | 37·25 |

The amount of digestion in these experiments is therefore very nearly the same for each kind of meat, and is proportional to the amount of matter capable of digestion present as measured by the quantity of nitrogen existing in each kind. These slight variations in the amount of nitrogen might be due to the variations in the amount of fat existing in the samples examined, and would point to the conclusion that the particular legs of Australian and New Zealand mutton used for these experiments were slightly fatter than the leg of Scotch mutton.

"The freezing of the foreign meat seems to have slightly reduced the amount of water present in the minced meat, so that the foreign meat weight for weight has a greater amount of food constituents if the fat be taken into account."

Little comment is necessary on the above results. The difference between the English and foreign meat is so slight as again to suggest the probability that, had all the samples been English or all foreign, taken from different animals, the results could hardly have worked out more evenly. There seems little doubt that the freezing process reduces the amount of water, as was shown by the former experiments in the lessened amount of evaporation in the oven, of the foreign meat; but, if König's table be compared, it will be seen how very slight a variation is revealed. As regards the proportion of nitrogen contents in the three samples, it may be observed that, even if foreign meat were inferior in nitrogen to the extent indicated above on an average, it would hardly be appreciable in a diet. The conclusion arrived at by Dr. Rideal, however, that the variation in the amount of nitrogen is due to the accidental character of the samples, is borne out by the fact that the Canterbury mutton shows a greater deficiency than the Australian, although the latter is always sent over very hard frozen, and is not, as may be seen by the price, considered by any means of equal value in the market to the former. If the freezing process is responsible for the reduction of the nitrogen, it is hard to understand why the Canterbury mutton, which is preserved in a temperature only just below freezing-point, should have lost more than the other.

The artificial digestion tests are among the most interesting which Dr. Rideal has carried out in this connection. The digestibility of the meat is all important from the invalid's standpoint, and remembering that foreign meat is very commonly indeed used for the patients' diets, it is very reassuring to find that the freezing process does not to any appreciable extent render it less easy of assimilation or constitute a drawback to its use for persons of feeble digestive powers.

That foreign meat is not unpalatable may be judged from the fact that it is consumed in more than one institution and pronounced excellent by persons who never doubt that it is home-grown and killed, while experiments made on those who believe their judgment in the matter to be reliable have proved that after cooking the process of discrimination is never a certainty.

